# LCIG in treatment of non‐motor symptoms in advanced Parkinson’s disease: Review of literature

**DOI:** 10.1002/brb3.1757

**Published:** 2020-07-16

**Authors:** Walaa A. Kamel, Jasem Y. Al‐Hashel

**Affiliations:** ^1^ Neurology Department Ibn‐Sina Hospital Kuwait City Kuwait; ^2^ Neurology Department Faculty of Medicine Beni‐Suef University Beni‐Suef Egypt; ^3^ Department of Medicine Faculty of Medicine Kuwait University Kuwait City Kuwait

**Keywords:** carbidopa, intestinal gel, levodopa, nonmotor, Parkinson's disease

## Abstract

**Background:**

For managing nonmotor symptoms (NMS) in advanced Parkinson's disease (PD), levodopa–carbidopa intestinal gel (LCIG) infusion is of interest as it shows lesser plasma fluctuations of both drugs as compared to oral levodopa–carbidopa (LC).

**Objectives:**

To highlight LCIG effect in NMS among advanced PD patients and appraise the currently available literature.

**Methods:**

PubMed screening (till 2020) of 184 articles was done, of which 51 were selected. Among them, 23 original articles relevant to the research question were included, of which 6 were then excluded after careful reading of full articles. The 17 relevant studies of the review provide Grade C level of evidence of efficacy.

**Results:**

LCIG is beneficial in improving or relieving various NMS especially (mood, cognition/memory, sleep, gastrointestinal symptoms, urinary symptoms, and quality of life questionnaires) in patients with advanced PD. Amelioration of motor functions or direct relations may lead to improvement in NMS PD patients using LCIG. Adverse events noted in patients treated with LCIG include pneumoperitoneum, abdominal pain, stoma infection, reversible peripheral neuropathy, local tube problems, impulse control disorder, and weight loss. Serious adverse events were mostly found to be unrelated to LCIG.

**Conclusions:**

LCIG provides an uninterrupted intestinal levodopa infusion by percutaneous endoscopic gastrojejunostomy (PEG‐J). It effectively decreases plasma fluctuations of levodopa and reduces motor instability and NMS burden in advanced PD. However, adequate dose modification and individualization of therapy are essential for optimal effect.

## Graphical abstract

LCIG provides an uninterrupted intestinal levodopa infusion. It effectively decreases plasma fluctuations of levodopa and reduces motor instability and NMS burden in advanced PD. However, adequate dose modification and individualization of therapy are essential for optimal effect.

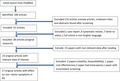

## 1 INTRODUCTION

Parkinson's disease (PD) is one of the most common neurodegenerative disorders that majorly affect elderly individuals. Degeneration of dopamine‐producing brain cells (owing to their high energy demands) leads to the development of PD (Mamelak, 2018; Benamer, de Silva, Siddiqui, & Grosset, 2008). According to the Global Burden of Diseases, Injuries and Risk Factors Study (2016), globally 6.1 million people suffered from PD (Collaborators GBDPsD, 2018). The primary motor symptoms observed in PD patients include tremors, bradykinesia (slow movement), muscle stiffness (rigidity), and postural instability. Apart from motor symptoms, nonmotor symptoms (NMS) associated with PD are depression, anxiety, sleep disturbances, constipation, fatigue, cognition loss, urinary complications, and impairment of olfaction (Al‐Mubarak et al., 2015; Luquin, Kulisevsky, Martinez‐Martin, Mir, & Tolosa, 2017; Shrestha et al., 2017). Advanced PD is characterized by further progression in motor and functional deterioration, and worsening of motor and NMS complications. For patients with advanced PD, conventional therapy may not be enough for the management of the condition (Al‐Mubarak et al., 2015; Luquin et al., 2017; Shrestha et al., 2017). Various alternative therapies known as “device‐aided treatments” are available for the management of motor symptoms; however, NMS are not focused by the presently available therapies (Kelberman & Vazey, 2016; Luquin et al., 2017).

Nonmotor symptoms may result from dopaminergic or nondopaminergic neurotransmissions, thus cannot be completely improved with dopamine replacement therapy alone (such as levodopa alone), the gold standard for treatment of PD (Tomlinson et al., 2010; Tsui & Isacson, 2011). Moreover, higher doses of levodopa may complicate PD and lead to NMS (Tomlinson et al., 2010). Furthermore, there is a need to resolve the significant challenge associated with oral levodopa–carbidopa (L‐C), that is, the variability and fluctuations in levodopa–carbidopa plasma concentration. This has encouraged researchers to evaluate the efficacy and safety of levodopa–carbidopa intestinal gel (LCIG) infusion.

Studies suggest that both drugs (levodopa and carbidopa) when given as infusion gel show minor variation and fluctuations in plasma concentration compared to levodopa–carbidopa–oral and are therefore of great importance in improving NMS associated with PD (Othman, Rosebraugh, Chatamra, Locke, & Dutta, 2017).

Thus, to highlight the effects of LCIG in treatment of NMS among advanced PD patients, we conducted this study appraising the currently available literature for identifying gaps in the available evidence.

## 2 METHODS

### 2.1 Ethical compliance statement

The authors confirm that the approval of an institutional review board and informed patient consent was not required for this work.

The objective was addressed using a structured, evidence‐based, critically appraised topic (CAT) format. This includes structuring a focused and answerable clinical question, search strategy, identifying and evaluation of evidence, reporting and interpretation of results, and bottom‐line clinical conclusions.

Structured Question: Is LCIG effective for the treatment of NMS in advanced PD?

### 2.2 Search strategy

We searched the electronic database PubMed till 2020 to identify relevant studies performed using the search terms: levodopa AND carbidopa AND Parkinson's disease AND non‐motor; levodopa AND carbidopa AND Parkinson's disease AND non‐motor symptom; levodopa AND carbidopa AND Parkinson's disease AND non‐motor symptoms AND efficacy. Original research articles, case reports, and systematic reviews were considered for inclusion in the present CAT. Further, all animal studies, letters to editors, and narrative reviews were excluded. Only articles published in the English language were considered for inclusion. No time limit was applied for searching articles.

We have classified the NMS of PD from the selected studies into 6 categories, viz. nervous system symptoms (mood changes, anxiety, irritability, akathisia, sleep disturbance, insomnia/difficulty in sleep, nightmares, daytime sleepiness, day time fatigue, psychiatric symptoms, excessive sleep, restless leg syndrome, attention, emotion/emotional well‐being, memory/cognition, sadness, and communication), cardiovascular system symptoms (palpitations, cardiovascular symptoms, tightness sensation), gastrointestinal tract symptoms (constipation, nausea, vomiting, drooling/ dribbling, and other gastrointestinal symptoms), systemic symptoms (weight loss, fatigue, muscle cramps, pain, diffuse pain, excessive sweating/hyperhidrosis), urinary symptoms (unspecified urinary symptoms), reproductive system symptoms (sexual function; Antonini et al., 2017; Antonini, Yegin, Preda, Bergmann, & Poewe, 2015; Bellante, Dethy, & Zegers de Beyl, 2016; Blaise, Baille, & Carrière, 2020; Buongiorno et al., 2015; Cáceres‐Redondo, Carrillo, & Lama, 2014; Chang et al., 2016; De et al., 2017; Fasano, Ricciardi, Lena, Bentivoglio, & Modugno, 2012; Honig et al., 2009; Krüger et al., 2017; Lopiano, Modugno, & Marano, 2019; Santos‐Garcia et al., 2012; Standaert et al., 2017; Valldeoriola et al., 2016; Wang, Li, & Chen, 2018; Wetmore et al., 2019; Zibetti, Rizzone, et al., 2013).

## 3 RESULTS

### 3.1 Identified evidence

**FIGURE 1:**
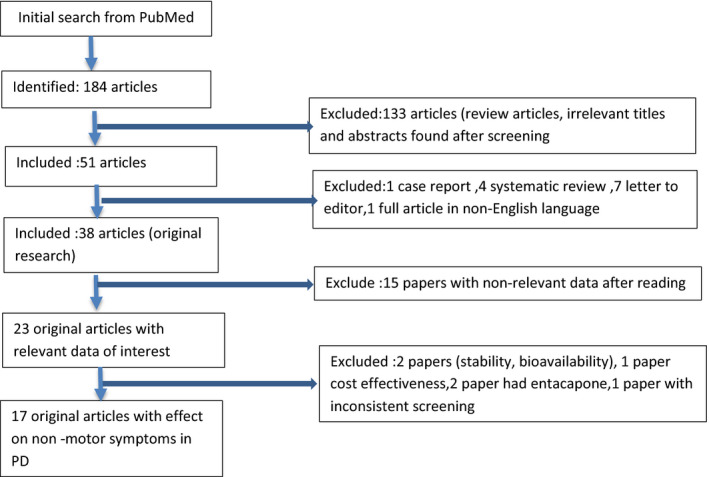
Flowchart representing the inclusion of studies with evidence related to nonmotor symptoms

Our literature search resulted in 184 articles on PubMed. Of these, 51 articles were selected, and 133 were excluded. Original research articles were preferred over other study types. Review articles were not excluded from the search initially to identify relevant articles from the bibliography of these articles. The titles and abstracts of all the articles were screened to identify the relevant ones. Full texts of all potentially relevant articles were procured for further screening. The bibliographies of these articles were examined for any additional pertinent citations. After going through the shortlisted 51 articles, 38 were original research articles, one was a case report, four were systematic reviews, seven were letters to the editor, and one full article was in non‐English language. Of these 38, 23 original articles, providing data relevant to the research question, were included. Among them, 17 papers commented on effect of LCIG treatment on NMS in PD patients. The excluded 6 articles focused on the stability of plasma levodopa levels and bioavailability (two articles), while one reported cost‐effectiveness analysis. Another two articles discussed the beneficial effects of the addition of entacapone to levodopa and carbidopa therapy. One article was excluded due to inconsistent screening of NMS, hence excluded. The detailed study selection criteria are illustrated in Figure 1.

### 3.2 Evaluation of evidence

Selected articles demonstrated the effect of LCIG on patients with PD, wherein an improvement in NMS was observed in most of the articles. These studies demonstrated that the intestinal gel formulation helps in bypassing gastric emptying and overcoming fluctuation in plasma drug levels. The study characteristics of all the articles included in the present CAT are presented in Table 1.

**TABLE 1:** Study characteristics

Study	Study type	Study population	Patients	Nonmotor symptoms evaluated	Evaluation Tests for corresponding nonmotor symptom	Results
Bellante et al. (2016)	Prospective, observational study	Idiopathic Parkinson's disease	10	Mood	17‐Items Hamilton Depression Scale (HAM: range 0–52; a decrease of the scale indicates an improvement of mood)	Improvement was observed in 9 of 10 patients. Improvement was significant after 2 months of treatment (*p < *.01) than after 1 month of treatment (*p = *.1)
				Anxiety	Hospital anxiety And Depression Scale (HAD: range 0–42; a decrease of the scale indicates an improvement of mood and anxiety)	Improvement observed in 7 of 10 patients. Significant improvement after 1‐month treatment (*p = *.06). At 2 months, improvement was not significant (*p = *.19)
				Non‐motor symptoms	Non‐Motor Symptom Scale (NMSS: 0–480; a decrease indicates an improvement of NMS) and PD	NMSS improvement was observed in 9 of 10 patients, and the improvement was significant after both 1 month and 2 months of treatment (*p < *.01 for both)
				Sleep	(PDSS: range: 0–150; an increase indicates an improvement of sleep)	PDSS improved to a significant extent in 9 of 10 patients after 1 month of treatment (*p < *.05) while improvement was observed in all 10 patients after 2 months of treatment (*p < *.01)
Bohlega et al. (2015)	Prospective single‐ movement disorder center study	Parkinson's disease	20	Nonmotor symptoms	NMSS, and PDQ‐8 data pre‐LCIG and 6 months post‐LCIG were compared and mean percentage improvement in NMSS and PDQ−8 was calculated	Mean NMSS: pre‐LCIG was 237.1 ± 45.5; mean NMSS: 6 months post‐LCIG was 81.6 ± 25.7 (*p < *.001); mean percentage improvement in the NMSS, 6 months post‐LCIG was 65%
				Quality of life		Mean PDQ‐8: pre‐LCIG was 23.2 ± 4.4; mean PDQ‐8: 6 months post‐LCIG was 8.0 ± 3.5 (*p < *.001); mean percentage improvement in the PDQ‐8, 6 months post‐LCIG was 65.7%
						Majority (78.8%) of the patients developed at least one adverse event during the study period. However, majority of them were minor, device‐related, and do not impose any risk on patient's life
Buongiorno et al. (2015)	Observational, prospective, open‐label study	Parkinson's disease	72	Behavioral and mood disorders (anxiety, depression, and irritability)	Percentage decrease in patients from baseline to during last visit (LV) was compared	Baseline: 66% patients; during LV: 38% patients (*p* < .0001)
				Dysautonomia symptoms (hyperhidrosis)		Baseline: 60% patients, during LV: 33% patients (*p = *.0015)
				Sensory symptoms (painful paresthesia)		Baseline: 48% patients; during LV: 30% patients (*p < *.0001)
				Constipation		Baseline: 58% patients; during LV: 46% patients (*p < *.0001)
				Fatigue		Baseline: 51% patients; during LV: 36% patients (*p = *.0127)
				Pain		Baseline: 49% patients; during LV: 36% LV patients (*p = *.049)
				Insomnia		Significantly decreased 3 months after the LCIG (*p = *.0053)
				Nightmares hallucination or psychotic Dopamine dysregulation syndrome (DDS)		Significant decreased over the study (*p = *.0075). Number of patients with hallucination/psychosis at baseline (10), slightly increased during LV (13). Baseline (10) patients, LV (6)
				Cognition Depression Day time somnolence and restless leg syndrome (RLS) Orthostatic dysautonomic		Percentage of patients presenting symptoms of dementia did not differ along the study. Baseline: 58%, LV: 46% No difference between BL to LV in the percentage of patients. No difference. Twenty‐eight (28) patients discontinued the treatment (half of them withdrew within the first three months). Adverse effects included pneumoperitoneum (in 54% of the patients), abdominal pain (20%), and stoma infection (3%)
Fasano et al. (2012)	Retrospective, open‐label study	Advanced Parkinson's disease	14	Sleep/fatigue Daytime sleepines Fatigue Difficulty in sleep Restless leg syndrome (RLS)		Pre‐CIILG: 14.21 ± 9.28; Post‐CIILG: 9.93 ± 6.65; percentage of change: −30.2 (*p = *.0987) Pre‐CIILG: 2.79 ± 2.46; Post‐CIILG: 2.36 ± 2.37; percentage of change: −15.4 (*p = *.1894). Pre‐CIILG: 4.21 ± 3.47; Post‐CIILG: 3.43 ± 2.90; percentage of change: −18.6 (*p = *.3437) Pre‐CIILG: 4.50 ± 3.70; Post‐CIILG: 2.21 ± 2.01; percentage of change: −50.8 (*p = *.0535) Pre‐CIILG: 2.71 ± 3.10; Post‐CIILG: 1.93 ± 2.16; percentage of change: −28.9 (*p* = .3701)
				Cognitive and neuropsychiatric	Frontal Assessment Battery (FAB), Neuropsychiatric Inventory) NPI, and Mini‐Mental State Examination (MMSE)	Significant improvement only in NPI:pre‐LCIG = 41.15 ± 30.74, post = 27.38 ± 23.01;‐ 33.5%; *p* = .0150
				Depression Delusions Excessive sweating Sleep Emotion/behavior ICD Quality of life (QoL)	NMSS domain 3 NMSS domain 4 NMSS domain 9 PDSS Unified PD‐Rating Scale (UPDRS‐I) Questionnaire for ICD in PD(QUIP) PDQ	pre = 5.79 ± 3.51, post = 3.93 ± 3.15; −32.1%; *p* = .0274. 4.29 ± 4.53 versus 1.79 ± 1.89; −58.3%; 0.0292. 4.21 ± 3.47 versus 2.93 ± 2.64; −30.5%; 0.0097. 39.08 ± 8.58 versus 33.46 ± 9.21; −14.4%; 0.0079. Thought disorders: 2.29 ± 0.99 versus 1.71 ± 0.99; −25%; 0.0281. Depression: 2.43 ± 0.065 versus 1.71 ± 0.73; −29.4%; 0.0003. Motivation/initiative: 2.21 ± 0.97 versus 1.79 ± 0.97; 19.3; 0.0047. 0.62 ± 0.51 versus 0.29 ± 0.47; −53.6%; 0.0262. 18.1 ± 6.6 versus 16.7 ± 6.0; −7.7%; 0.285 marginally modified
Valldeoriola et al. (2016)	Observational, multicenter, cross‐ sectional, retrospective study	Advanced Parkinson's disease	177	Dizziness Fatigue during daytime Flat mood Falling asleep during daytime Insomnia Sadness	Percentage of patients with NMS symptom improvement was calculated using 31‐item NMS questionnaire that included 30 questions of NMSS plus impulse behavior	59.7% patients 57.5% patients 56% patients 52.6% patients 52.3% patients 50.9% patients
Zibetti et al. (2013)	Prospective study	Parkinson's disease	12	Total PDSS−2 (Parkinson's Disease Sleep Scale)	Baseline and 2‐ to 4‐month follow‐up PD‐Sleep‐Scale version‐2 (PDSS‐2) total score, subscores for “Disturbed sleep,” “PD symptoms at night” and ESS score were compared	Baseline = 34; follow‐u*p = *20.9; % change 34%, *p* = .005
				Disturbed sleep		Baseline = 13.2; follow‐u*p = *8.7; % change 31.7%, *p* = .005
				PD symptoms at night		Baseline = 9.5; follow‐u*p = *5.6; % change 30.2%, *p* = .046
				ESS (Epworth Daytime Sleepiness Scale)		Baseline = 7.7; follow‐u*p = *5.3; % change 31.2%, *p* = .033
				Motor symptoms At night		Subjective measures of sleep quality and daytime sleepiness improved in patients with advanced PD and treated with LCIG infusion. Baseline = 11.3; Follow up = 6.6; % Change 27.9%, *p* = .017
Slevin et al. (2015)	Phase 3 Open‐label extension of the doubleblind pivotal study	Advanced Parkinson's disease	62	PDQ‐39 summary index	Mean change in PDQ‐39 SI, EQ‐5D summary index, EQ‐5D VAS score and ZBI score was measured for ‘Continuing LCIG’ group and ‘LCIG naïve’ group from baseline to final visit	Continuing LCIG = 1.5; LCIG naïve = −3.5
				EQ−5D summary index		Continuing LCIG = −0.009; LCIG naïve = −0.006
				EQ−5D VAS score		Continuing LCIG = −0.9; LCIG naïve = 4.5
				ZBI score		Continuing LCIG = 1.1; LCIG naïve = −1.8
Santos‐Garcia et al. (2012)	Prospectively open‐label	Advanced Parkinson's disease	11	PDQ‐39 SI Emotional well‐being	Improvement in total PDQ‐39SI and emotional well‐being was studied after short‐ and long‐term exposure	Improved 6 months after beginning with DLI (29.7 ± 8.6, *p* = .008) and at the last visit 34.8 ± 11.2, *p* = .008) compared with baseline (55.6 ± 11.5) Improved 6 months after beginning with DLI (31 ± 20.9) and at the last visit (36.4 ± 15.8) compared with baseline (60.7 ± 21.4) *p* = .008
Palhagen et al. ( 2012)	An interin 12 M analysis is a part of Open‐label, observational, prospective study	Parkinson's disease	27	Total UPDRS total score (mean ± *SD*)	Total UPDRS total scores and Total PDQ‐39 scores were assessed at baseline, 3 months after surgery, and then every 3 months	LCIG provides functional improvement beginning at first visit which sustained for 12 months. Baseline = 52.1 ± 16.1, *N* = 27; month 0 (first visit, at least 3 months after permanent LCIG) = 43.1 ± 16.7, *N* = 27 (*p = *.003); month 12 = 42.5 ± 22.6, *N* = 25 (*p = *.017). UPDRS‐I (mentation, behaviour, and mood) showed little change
				Total PDQ‐39 score (mean ± *SD*)		Baseline = 33.6 ± 10.8, *N* = 27; month 0 = 27.1 ± 11.8, *N* = 27 (*p = *.001); 12 months = 28.8 ± 12.8, *N* = 23 (*p = *.126)
						Total number of SAE reported was 43 in 17 patients. 37% (16) of these SAEs were unrelated to LCIG. The remaining 27 were distributed in 13 patients. Two patients terminated the study after month 0 because of adverse effects
Antonini et al. (2015)	Prospective, noninterventional study	Advanced Parkinson's disease	375	NMS	NMS and PDQ‐8, EQSD scores were measured at baseline, and at M6 and M12 after LCIG	Total NMSS scores significantly improved at M6, 12 *p* = .0001, 0.0014 Significant improvements of NMS were observed up to M12 in 3 out of the 9 NMSS domains: At M 12, domain 2 (sleep/fatigue): _7.5 ± 13.1 (*p = *.0001). At M 12, domain 6 (gastrointestinal tract): _2.6 ± 7.1 (*p = *.0096). At M 12, domain 7 (urinary): _2.8 ± 8.7 (*p = *.0199). At M6, domain 3 (mood/cognition): _4.1 ± 16.7 (*p = *.0426)
				PDQ‐8 scores		In 3 out of the 8 PDQ‐8 items significant QoL improvements were observed at M12: At M 12, item 1 (difficulty getting around in public places): _0.5 ± 1.3 (*p = *.0074)
				EQ‐SD,VAS		At M12, item 3 (felt depressed): _0.4 ± 1.4 (*p = *.0372) At M12, item 8 (embarrassed by having PD): _0.5 ± 1.6 (*p = *.0312) At M6, item 7 (painful muscle cramps and pains): _0.5 ± 1.3 (*p = *.0031) At M6 by + 0.12 ± 0.35 (*p* = .0076), M12 by + 0.17 ± 0.25 (*p* = .0001) 5% of patients registered adverse drug reaction leading to LCIG treatment discontinuation. The most common side effects were weight loss and abdominal pain (5.6% and 3.1% respectively)
Standaert et al. (2017)	Open‐label phase 3b study	Advanced Parkinson's disease	39	Total NMSS	Least‐squares mean change from baseline at week 12 and week 60 were measured	Baseline = 48.3 ± 35.6; Week 12 = 17.6 ± 3.6 (*p=*<0.001); Week 60 = 11.8 (*p = *.004)
				Sleep/fatigue		Baseline = 11.6 ± 9.2; Week 12 = 6.0 ± 1.2 (*p=*<0.001); Week 60 = 5.4 ± 1.3 (*p < *.001)
				Attention/memory		Baseline = 4.6 ± 6.4; Week 12 = 2.1 ± 0.8 (*p = *.010); Week 60 = 2.2 ± 0.9 (*p = *.013)
				Gastrointestinal tract		Baseline = 5.3 ± 6.1; Week 12 = 2.0 ± 0.6 (*p = *.001); Week 60 = 1.9 ± 0.7 (*p = *.006)
				Sexual function		Baseline = 2.7 ± 3.6; Week 12 = 1.8 + 0.4 (*p=*<0.001); Week 60 = 1.1 + 0.5 (*p = *.021)
				Miscellaneous		Baseline = 8.3 ± 9.4; Week 12 = 3.4 + 1.0 (*p = *.001); Week 60 = 3.4 + 1.1 (*p = *.003)
				PDQ39SI		Baseline = 34.7 ± 13.0; Week 12=‐ 11.2 + 2.8 (*p < *.001); Week 60=−10.2 + 2.6 (*p < *.001)
						Adverse events were reported in 95% of the patients. 5 patients (13%) discontinued treatment due to AE, of which 4 were considered to be related to LCIG
Honig et al. (2009)	Prospective open‐label observational study	Advanced idiopathic Parkinson's disease	22	Total NMSS	Scores of applied measures from baseline were compared with 6‐ month follow‐up period score	Baseline = 89.9; follow‐u*p = *39.4 (*p = *.0001)
				Cardiovascular		Baseline = 2.9; follow‐u*p = *0.5 (*p = *.0004)
				Sleep/fatigue		Baseline = 18.1; follow‐u*p = *6.8 (*p = *.0001)
				Attention/memory		Baseline = 7.3; follow‐u*p = *4.0 (*p = *.002)
				Gastrointestinal tract		Baseline = 10.0; follow‐u*p = *3.8 (*p = *.0003).
				Urinary		Baseline = 11.4; follow‐u*p = *4.8 (*p = *.002)
				Miscellaneous (including pain and dribbling)		Baseline = 14.1; follow‐u*p = *6.4 (*p = *.0004)
				PD‐Sleep Scale		Baseline = 86.0; follow‐u*p = *114.5 (*p = *.002)
				PDQ‐8		Baseline = 44.2; follow‐u*p = *20.7 (*p = *.0003)
Krüger et al. (2017)	Prospective observational multicentre study	Advanced PD	64	NMS	NMSS and subdomains UPDRS part II, III, IV, ADL	Significant improvement from BL in QOL,NMSS at all points (*p* < .001 for all)specifically,patients manifested significant improvement in mean change from BL at every study visit in 5 of 9 NMSS domains (sleep/fatigue,mood/cognition gastrointestinal,urinary,and miscellaneous)
				PDQ	PDQ‐8 SI	Significant mean decrease from BL PDQ‐8 summary index score at all time points *p* < .0001 at M3,6; *p* < .001 at M12 and final
Wetmore et al. (2019)	Data are derived from ADEQUA study	Advanced PD on LCIG	61	Apathy	Apathy scale	Non‐significant improvement between BL and follow up visit *p* = .46(BL = 11.69 ± 6.67 versus 12.34 ± 6.52)
				Depression	Beck Depression Inventory (BDI‐II)	Significant improvement *p* = .0017 (BL 18.45 ± 9.71 versus 12.64 ± 10.31
				Anxiety	Beck Anxiety Inventory (BAI)	Significant improvement *p* = .0003 (BL = 20.12 ± 9.72 versus 13.60 ± 10.39)
				Fatigue	Parkinson fatigue scale(PFS−16)	Significant improvement *p* = .0001 (BL = 60.17 ± 11.48 versus 49.60 ± 14.40)
				NMS	NMSS	Significant improvement *p* < .0001 (BL = 83.83 ± 33.35 versus 48.13 ± 29.79)
				PDQ	PDQ‐39	Significant improvement *p* < .0001(BL = 46.74 ± 13.59 versus 33.66 ± 16.87)
Cáceres‐Redondo et al. (2014)	Prospective observational study	Advanced PD	29	Cognition	MMSE, the Mattis Dementia Rating Scale DRS (attention, frontal executive, visuospatial function, and memory), NPI‐Q	9/16 patients at follow‐up visit, were adequately assessed for cognition for the group of 4 patients who developed pro‐PDD total MMSE decreased from 22.2 ± 3.0 at BL to 17.8 ± 3.7 at follow‐up *p* > .05, DRS total score decreased 111.2 ± 19.5 at BL to 92 ± 14.3 *p* > .05
				NMS	NMSS	Total MMSE score in the remaining group who did not develop dementia decreased from 26.6 ± 2.4 at baseline to 25.4 ± 2.3 at follow‐up *p* > .05 and total DRS score decreased from 135.8 ± 3.7 at baseline to 133.6 ± 5.0 at follow‐up *p* > .05 A statistically significant beneficial effect was observed for (sleep/fatigue and gastrointestinal *p* < .05) and for the total score of this scale, remaining 6 categories (mood, cognition, cardiovascular, perception/hallucination, attention memory, urinary, and miscellaneous) showed a trend for improvement except for sexual function
Lopiano et al. (2019)	The GREENFIELD study,observational prospective and retrospective multicenter study	Advanced Parkinson's disease	145	Mentation, behavior, mood Sleep Impulsive disorders Gait, fall QOL	UPDRS‐I PDSS−2 QUIP‐RS Gait Fall Questionnaire (GFQ) PDQ39	Baseline mean score (on) is 4.3 ± 3.08, V2 is 3.6 ± 2.71, V3 is 3.8 ± 2.68 *p* = .054 at V2 versus baseline, 0.055 at v3 versus baseline. Baseline mean score is 25 ± 1.04, V2 is 22.5 ± 9.9, V3 is 22.7 ± 10.1 *p* < .01 *P* value is < .05 for sexual behaviors, <.05 for eating, <.01 for medications Baseline mean score is 29.7 ± 13.3, V2 is 26.5 ± 13.1, V3 is 26.1 ± 12, *p* is < .05 at V2 versus baseline Baseline mean score is 72.3 ± 23.8, V2 is 64.7 ± 25.4, V3 is 67.3 ± 26.4 *p* is < .001, <.05. −27.6% patients experienced 1 or more SAE, 16.3% were related to PEG/J procedure or to device, in 8.3% the AEs lead to discontinuation, 9% deaths occurred
Chang et al. (2016)	Open‐label, prospective study	Advanced Parkinson's disease	15	Total PDQ‐39 Summary index Communication and emotion Cognitive impairment	Total PDQ‐39 Summary Index at baseline and at 6 and 12‐month follow‐up were compared	At 6 m: improved by 38.9 ± 36%; At 12 m:improved by 32.5 ± 35% At 12 months: improved by at least 28% At 6 months: improved at least,by 2.4 ± 102%;at 12 months improved by 7.3 ± 97%

Abbreviations: PD, Parkinson's disease; PDQ, Parkinson's Disease Questionnaire; SI, Summary Index; PDSS, Parkinson's Disease Sleep Scale; ESS, Epworth Daytime Sleepiness Scale; EQ‐5D, EuroQol‐5 dimension; VAS, Visual Analogue Scale; ZBI score, Zarit Burden Interview; UPDRS, Unified Parkinson's Disease Rating Scale; *SD*, standard deviation; NMS, nonmotor symptom; NMSS, Non‐Motor Symptom Score; HAM, Hamilton Depression Scale; HAD, Hospital Anxiety And Depression Scale; LCIG, Levodopa–carbidopa intestinal gel; LV, Last visit; CIILG, continuous infusion of intrajejunal levodopa/carbidopa gel; M, month; DLI, duodenal levodopa infusion; *N*, number; GFQ, Gait Fall Questionnaire; MMSE, Mini‐Mental State Examination; QUIP, Questionnaire for ICD in PD; BDI, Beck Depression Inventory; BAI, Beck Anxiety Inventory; PFS, Parkinson's Fatigue Scale; DRS, The Mattis Dementia Rating Scale; RLS, restless leg syndrome; FAB, Frontal Assessment Battery; SAE, serious adverse events; NPI, Neuropsychiatric Inventory; DDS, dopamine dysregulation syndrome.

### 3.3 Nervous system (CNS) symptoms

Among the NMS of the CNS, the impact of LCIG on mood changes was reported in nine studies. Of these, Valldeoriola et al. (2016) evaluated the effect of LCIG on both motor and nonmotor symptoms in 177 patients and showed that flat mood improved in 99 (56%) patients with LCIG. Bellante et al. (2016) in their prospective, observational study reported mood improvement in nine of ten study patients, which were significant after two months of treatment with LCIG (*p* < .01). NMSS domain 3 for depression was improved in a study by Fasano et al. (2012) (*p* = .0274) and by UPDRS‐I (*p* =0 .0003). An improvement in Beck Depression Inventory II (BDI) was reported between baseline and follow‐up (*p* = .0017; Wetmore et al., 2019).

An improvement in anxiety was reported in three of 17 studies. Bellante et al. (2016) observed improvement in seven of ten patients, with significant improvement after one month of treatment (*p* = .06). A longitudinal analysis of 53 patients showed significant improvement in Beck Anxiety Inventory (BAI) between baseline and follow‐up visits (Wetmore et al., 2019).

Emotional well‐being showed an improvement to a considerable extent in one study (Santos‐Garcia et al. (2012). Valldeoriola et al. (2016) demonstrated improved communication and sadness after LCIG therapy, improvement in sadness was reported among 50.9% patients. Communication and emotion improved at 12 months by at least 28% (Chang et al., 2016). A nonsignificant improvement in Apathy Scale was shown between baseline and follow‐up (*p* = .46; Wetmore et al., 2019).

Psychiatric symptoms were significantly improved as revealed by the significant reduction of UPDRS‐I, Neuropsychiatric Inventory (NPI), Questionnaire for ICD in PD (QUIP), and specific items of NMSS, overall there was a significant improvement of depressive symptoms and psychiatric SE caused by dopamine agonist (DA) (i.e. delusions and ICD; Fasano et al., 2012). As per a study by Buongiorno et al. (2015), number of patients with hallucination/psychosis at baseline slightly increased during treatment from 10 increased to 13.

Impulsive disorders improved when assessed by Questionnaire for Impulsive Compulsive Disorders in PD‐Rating Scale (QUIP‐RS) in a study by Lopiano et al. (2019), *p* < .05 for sexual behavior, eating, and <0.01 for medications and by Fasano et al. (2012), *p* = .0262.

Sleep disturbance was improved significantly in five studies when were assessed by NMSS domain 2 (Antonini et al., 2015; Cáceres‐Redondo et al., 2014; Honig et al., 2009; Standaert et al., 2017; Wetmore et al., 2019). Parkinson's Disease Sleep Score (PDSS) improved in four studies (Bellante et al., 2016; Fasano et al., 2012; Wetmore et al., 2019; Zibetti, Rizzone, et al., 2013). As per a retrospective, open‐label study by Fasano et al. (2012) the percentage of change in difficulty in sleep before and after LCIG therapy was −50.8% (*p* = .0535; Bellante et al., 2016).

A prospective study conducted by Zibetti, Rizzone, et al. (2013) showed 31.7% change in sleep disturbance from baseline to follow‐up at 2–3 months. In an observational, prospective, open‐label study by Buongiorno et al. (2015), a significant (*p* = .0053) decrease in insomnia, three months after the LCIG, was observed. Another observational, multicenter, cross‐sectional, retrospective study conducted by Valldeoriola et al. (2016) showed insomnia improvement in 52.3% of patients. Buongiorno et al. (2015) reported that nightmares significantly decreased over the study duration (*p* = .0075). Significant improvement in follow‐up visits compared to baseline was observed in a prospective population for quality of sleep assessed by Parkinson's Disease Sleep Scale (PDSS‐2), *p* value < .01(Wetmore et al., 2019). Daytime sleepiness showed an improvement in two of 17 studies. As per Fasano et al. (2012) the percentage of change before and after LCIG therapy was −15.4% (*p* = .1894). Zibetti, Rizzone, et al. (2013) showed improvement in daytime sleepiness from baseline to follow‐up of two to four months by 31.2%. 19 falling asleep during day time improved in 52.6% of patients.

In addition to previous studies with subjective scales, further research found two papers with objective scales (polysomnography, PSG; De Fabregues, Ferré, Romero, Quintana, & Álvarez‐Sabin, 2018; Zibetti, Romagnolo, & Merola, 2017). An open‐label pilot study with a sample size limited to 11 patients that examined polysomnographic characteristics in PD patients on a stable LCIG dose, improvement of subjective sleep quality, PSG showed a reduction of the number of awakenings in sleep, a trend toward a lower apnea–hypopnea index, and no change in sleep latency, total sleep time, and sleep efficiency (Zibetti et al., 2017).

However, the results of a study conducted by De Fabregues et al. (2018) showed that the treatment with LCIG infusion was not associated with a significant amelioration of sleep quality, the overall quality of sleep in those patients was poor, but it was not found to be worsened by LCIG.

Valldeoriola et al. (2016) showed an improvement in daytime fatigue in 57.5% patients. Dizziness improved among PD patients on LCIG as reported in one study, wherein improvement was observed in 59.7% of the patients after receiving treatment (Valldeoriola et al., 2016).

Furthermore, restless leg syndrome improved in one of the included studies, wherein a 28.9% decrease was observed after LCIG therapy, although the change was not significant (*p* = .3701; Fasano et al., 2012) No difference was noted by Buongiorno et al. (2015) from baseline to last visit in the percentage of patients.

Six studies assessed memory/cognition; 2 studies showed no worsening of cognitive functions (Buongiorno et al., 2015; Fasano et al., 2012). In a series of patients, Mini‐Mental State Examination (MMSE) was assessed as a screening measure of global cognitive functioning; the Mattis Dementia Rating Scale (DRS) assessed four cognitive domains: attention, visuospatial functions, frontal executive and memory, and a considerable percentage (25%) of subjects developed a significant deterioration of cognitive functions over time, especially in executive functions and probably reflects the nature of PD (Cáceres‐Redondo et al., 2014). Chang et al. (2016) showed cognitive improvement by 2.4 ± 102% at 6‐month follow‐up and by 7.3 ± 97% at 12‐month follow‐up when compared to baseline. Significant improvement of mood/cognition was noted when assessing NMSS domain 3 (*p* = .0426, *p* < .001, respectively; Antonini et al., 2015; Krüger et al., 2017). Two studies reported that patients who could not be attentive prior to therapy showed an improvement (Honig et al., 2009; Wang et al., 2018).

As cognition is almost undeveloped in our search strategy for NMS, separate search for effect of LCIG on memory and cognition found 4 studies that showed improvement (De et al., 2017; Merola, Espay, & Romagnolo, 2016; Valldeoriola et al., 2017; Zibetti, Merola, & Ricchi, 2013b) In a study conducted by Zibetti, Merola, et al. (2013), up to 41% of LCIG‐treated patients showed impaired memory and cognitive flexibility after 3 years of follow‐up and it could not be excluded that cognitive changes were related to disease progression.

In a retrospective analysis of five‐year data from patients at similar baseline disability, treated with subthalamic nucleus deep brain stimulation (STN‐DBS), LCIG, oral medical therapy (OMT), patients were classified at baseline assessment and follow‐up visits as PD‐mild cognitive impairment (PD‐MCI) and PD‐dementia (PD‐D) according to different neuropsychological assessment including MMSE; PD‐D developed in 25% LCIG and 25% in OMT groups (from 0% baseline); PD‐MCI was ascertained in 30% and 40%, respectively (from a 5% and 10% baseline PD‐MCI prevalence; Merola et al., 2016).

Patients treated with LCIG may significantly improve some specific neuropsychological functions when compared with patients receiving STN‐DBS and with patients receiving conventional OMT after 1 year from the intervention (Krüger et al., 2017); after LCIG, there was an improvement in verbal memory, short‐ and long‐term attentional functions, voluntary motor control, phonetic verbal fluency, and naming; no statistical significant difference was found between baseline scores and after 6 months of treatment (De et al., 2017).

### 3.4 Cardiovascular system symptoms

Honig et al. (2009) in their prospective, open‐label, observational study demonstrated a decrease in cardiovascular system (CVS) symptom score from baseline (2.9) to six‐month follow‐up (0.5, *p* = .0004). A trend for improvement for CV domain of NMSS was shown in a study by Cáceres‐Redondo et al. (2014).

### 3.5 Gastrointestinal tract symptoms

Various studies showed improvement in gastrointestinal tract (GIT) symptoms, of which Buongiorno et al. showed a decrease in the number of patients with constipation from baseline (58%) to during the last visit (46%, *p* < .0001; Buongiorno et al., 2015).

Gastrointestinal (GI) symptoms improved in six studies (Standaert et al., 2017). A study by Honig et al. (2009) also showed GI symptom score decrease from baseline (10.0) to six‐month follow‐up (3.8, *p* = .0003).

The global long‐term registry on efficacy and safety of LCIG in patients with advanced Parkinson's disease in routine care (GLORIA Registry) in a prospective, noninterventional study evaluated the effect of LCIG in 375 patients with PD over a period of 24 months. GI symptoms improved significantly (−2.2 ± 7.3, 95% CI: −3.1, −1.2, *p* < .001) after LCIG therapy (Antonini et al., 2015, 2017). Standaert et al. (2017) reported improvement in GI symptoms from baseline (5.3 ± 6.1) to weeks 12 (2.0 ± 0.6, *p* = .001) and weeks 60 (1.9 ± 0.7, *p* = .006). Krüger et al and Cáceres‐Redondo et al showed a significant improvement in GIT symptoms (De Fabregues et al., 2018; Zibetti, Rizzone, et al., 2013).

Drooling/dribbling improved in one study by Honig et al. (2009) in terms of a decrease in the number of patients with symptoms and symptom score from baseline to six months, respectively.

### 3.6 Systemic symptoms

Thirty‐eight percent of patients included in the study conducted by Blaise et al. (2020) experienced a weight loss; about half of these lost more than 7% of their initial weight (Wang et al., 2018), between 6.7% and 24.3% of patients treated by LCIG experience weight loss (Antonini et al., 2017; De et al., 2017). However, these studies did not report data on enteral nutrition.

An improvement in fatigue was noted in three studies. An observational, prospective, open‐label study conducted by Buongiorno et al. (2015) showed a reduction in the percentage of patients with fatigue from baseline (51%) to last visit (36%) to a significant extent (*p* = .0127). The percentage of change in fatigue decreased by −18.6% (*p* = .3437) in a retrospective study by Fasano et al. (2012) Parkinson's Fatigue Scale (PFS‐16) was significantly improved between baseline and follow‐up after 6 months in the study by Wetmore et al. (2019).

The effect of LCIG on pain in PD patients was evaluated in three of 17 studies included in the CAT. As per a study by Honig et al. (2009), an improvement in miscellaneous symptoms including pain was noted (*p* = .0004). Buongiorno et al. (2015) showed a reduction in the number of patients with painful paresthesia from baseline (48%) to last visit (30%, *p* < .0001).

The GLORIA registry showed improvement in muscle cramps and pain with LCIG over a period of 24 months (−0.3 ± 1.4, 95% CI: −0.5, −0.1, *p* = .002; Antonini et al., 2017).

Excessive sweating/hyperhidrosis improved in two of the 17 studies included. A study by Fasano et al. (2012) reported a significant improvement in excessive sweating as assessed by NMSS domain 9 (*p* = .00097). Another observational, prospective study by Buongiorno et al. (2015) demonstrated a significant decrease (*p* = .0015) in the percentage of patients with hyperhidrosis from baseline (60%) to last visit (33%).

### 3.7 Urinary symptoms

Urinary symptoms improved in a total of three studies and a trend for improvement in one (Cáceres‐Redondo et al., 2014). While the GLORIA registry reported the effect of LCIG therapy on urinary symptoms at month 12 from baseline (−2.8 ± 8.7, *p* = .0199), the study did not report the effect on these outcomes at month 24 (Cáceres‐Redondo et al., 2014). Valldeoriola et al. showed a decrease in urinary symptom score from baseline (11.4) to follow‐up (4.8, *p* = .002; Valldeoriola et al., 2017) Krüger et al. (2017) showed a significant improvement in mean change from baseline at every study visit in urinary symptoms.

### 3.8 Reproductive system symptoms

In an open‐label phase 3b study by Standaert et al. (2017) sexual functions improved from baseline (2.7 ± 3.6) to week 12 (1.8 ± 0.4, *p* < .001) and week 60 (1.1 ± 0.5, *p* = .021).

### 3.9 Severity of NMS assessed with rating scales

Several rating scales are used to assess the severity of NMS symptoms of PD patients. Two studies reported improvement in total Non‐Motor Symptoms Scale (NMSS) score (Bellante et al., 2016; Cáceres‐Redondo et al., 2014), seven studies reported improvement in total Parkinson's Disease Questionnaire (PDQ) scores (Antonini et al., 2015; Chang et al., 2016; Lopiano et al., 2019; Palhagen et al., 2012; Santos‐Garcia et al., 2012; Slevin et al., 2015; Wetmore et al., 2019), and 4 studies reported improvement in both NMSS and PDQ scores (Bohlega et al., 2015; Honig et al., 2009; Krüger et al., 2017; Standaert et al., 2017).

A long‐term global study assessed the effectiveness of LCIG in 375 patients across 18 countries. The study reported significant improvement in UPDRS II, UPRDS III “On” scores, total NMSS, and PDQ‐8 from baseline to follow‐up at month 12 (*p* = .0107, *p* = .0128, *p* = .0014 and *p* = .0100; Antonini et al., 2015).

A statistically significant beneficial effect was observed for sleep/fatigue and gastrointestinal and for the total score of NMSS; the remaining six categories (mood/cognition, cardiovascular, perception/hallucination, attention/memory, urinary, and miscellaneous) showed a trend for improvement except sexual function (Cáceres‐Redondo et al., 2014). LCIG‐treated patients show significant improvement in mean changes from baseline at every study visit in 5 of 9 NMSS domains: sleep/fatigue, mood/cognition, gastrointestinal tract, urinary, and miscellaneous NMSS subscores (Krüger et al., 2017).

An interim 12‐month analysis as a part of an open‐label, observational, prospective study on LCIG in PD to investigate clinical and health‐related quality of life by UPDRS, PDQ‐39 at baseline, ≥3 m after surgery, and then every 3 m showed that UPDRS total scores and PDQ‐39 scores improved significantly throughout the year, and UPDRS‐I (mentation, behavior, and mood) showed little change over the study period (Palhagen et al., 2012).

A recent study showed significant improvement between baseline and follow‐up in NMSS, PDQ‐39, mean scores of NMSS at baseline, follow‐up visits are 83.83 ± 33.35, 48.13 ± 29.79 respectively with *p* value < .0001, mean scores of PDQ39 at baseline, follow‐up visits are 46.74 ± 13.59, 33.66 ± 16.87 respectively with *p* value < .0001 (Wetmore et al., 2019). A significant improvement compared to baseline (BL) was observed in the prospective population for quality of life assessed by PDQ39; mean scores are (72.3 ± 23.8, 64.7 ± 25.4, 67.3 ± 26.4, at baseline, V2, V3, respectively), *p* < .001 between V2 and BL, and *p* < .05 between V3 and BL (Lopiano et al., 2019).

The efficacy and safety of LCIG in 20 PD patients were assessed in a study using unified Parkinson's Disease Rating Scale (UPDRS III), NMSS and PDQ‐8. Pre‐LCIG, the mean UPRDS, mean PDQ‐8, and mean NMSS were 55.8 ± 11.7, 23.2 ± 4.4, and 237.1 ± 45.5, respectively. At 6 months, significant improvement was noted with all three rating scales: the mean UPRDS, mean PDQ‐8, and mean NMSS were 19.6 ± 8.4, 8.0 ± 3.5, and 81.6 ± 25.7 (*p* < .001), respectively (Bohlega et al., 2015).

Thus, various scales used to assess improvement in motor and nonmotor symptoms in patients using LCIG have shown favorable results.

### 3.10 Adverse events of LCIG

Various AEs found to occur in clinical studies include pneumoperitoneum, abdominal pain, stoma infection, gastrostomy, reversible peripheral neuropathy, local tube problems, ICD, weight loss, and worsening of dysphagia (Bohlega et al., 2015; Wetmore et al., 2019; Buongiorno et al., 2015; Antonini et al., 2015; Krüger et al., 2017; Valldeoriola et al., 2017). Serious adverse events were mostly found to be unrelated to LCIG (Palhagen et al., 2012; Wang et al., 2018) The details of the AEs reported in various studies are presented in Table 1.

A study assessing the long‐term response to LCIG (mean observation time of 22 months and a maximum of 48 months) reported that 28 patients discontinued the study with reasons being stated as inefficacy (*n* = 13) or AEs related to the drug (*n* = 8). The AEs reported were severe dyskinesias, symptomatic orthostatic hypotension, bothersome sleepiness, uncontrolled punding, and anorexia. The study showed a significant increase in the percentage of the day with dyskinesias after the treatment (30% before treatment versus 40% in LV, *p* = .019). But when analyzed by dividing the study population into two groups (group 1: less than 50% of the day with dyskinesia versus group 2: more than 50% of the day with dyskinesia), group 2 showed significant improvement in the percentage of day with disabling dyskinesias (*p* = .04). It is important to conduct more studies with various patient profiles to assess, which responds better to LCIG treatment (Buongiorno et al., 2015).

A study from the Middle East assessing the safety of LCIG in PD patients reported 78.8% of the patients to have developed at least one AE. The complications reported were stoma infection (*n* = 2); maculopapular rash (*n* = 1); pump replacement (*n* = 5) in lieu of breakage or malfunctioning; and tube replacement (*n* = 12) resulting from accidental tube dislocation/slippage outside the body, tube dislocation to the stomach, and tube blockage due to knot formation. However, these were minor device‐related AEs and not classified as serious (Bohlega et al., 2015).

One‐third of the patients assessed in a study by Krüger et al. (2017) experienced an AE possibly related to LCIG, as rated by the study investigator. Two patients (3.1%) died during the study, and causes of death were cardiac failure and sudden death; both deaths were deemed by the investigator as having no reasonable possibility of being related to LCIG; seven patients (11.1%) discontinued LCIG treatment because of AES (Krüger et al., 2017).

A recent meta‐analysis of 8 studies reported heterogeneity in nonserious adverse event (AE) (*I* ^2^ = 52%, *p* = .06), while no heterogeneity was reported in serious AE (*I* ^2^ = 0%, *p* = .76). No incident of death was reported in most of the included studies. However, one study reported four deaths (control, *n* = 2, and LCIG, *n* = 2). Investigator classified the relationship of death to study drug as unlikely related (*n* = 1) to medications, unrelated (*n* = 2), and possibly related (*n* = 1; cardiac arrest; Wang et al., 2018).

A retrospective analysis of data on AEs in patients treated with LCIG at a French university medical center showed that 90% of patients experienced at least one AES. Most of them were related to PEG‐J or affected the gastrointestinal tract, device‐related AES was frequent in 63.5% of patients, and dopa therapy‐related AES occurred in 48% of patients (Blaise et al., 2020).

### 3.11 Study limitations

The study included 17 research articles, out of which two were retrospective in nature (Fasano et al., 2012; Valldeoriola et al., 2016). Fourteen studies were prospective (Antonini et al., 2015, 2017; Bellante et al., 2016; Bohlega et al., 2015; Buongiorno et al., 2015; Cáceres‐Redondo et al., 2014; Chang et al., 2016; Honig et al., 2009; Krüger et al., 2017; Palhagen et al., 2012; Santos‐Garcia et al., 2012; Standaert et al., 2017; Wetmore et al., 2019; Zibetti, Rizzone, et al., 2013). One of the studies was both prospective and retrospective (Lopiano et al., 2019). Additionally, most of them included less than 30 patients (Bellante et al., 2016; Bohlega et al., 2015; Cáceres‐Redondo et al., 2014; Chang et al., 2016; Fasano et al., 2012; Honig et al., 2009; Palhagen et al., 2012; Santos‐Garcia et al., 2012; Zibetti, Rizzone, et al., 2013). None of the article was placebo‐controlled or compared LCIG to oral treatment (except in the study conducted by Krüger et al. (2017), patients in the standard of care (SOC) group were assessed regarding improvement in NMS; however, the small size of the group (6 patients) did not allow for statistical analysis. Thus, all these relevant studies provided Grade C level of evidence for LCIG efficacy.

## 4 CONCLUSIONS

The efficacy of levodopa‐carbidopa combination is well established for the treatment of PD. Long‐term use of oral therapy may cause various fluctuations in response leading to the motor as well as nonmotor complications. Various selected observational studies and clinical trial studies have supported the use of LCIG in improving NMS especially (mood, cognition, sleep, gastrointestinal, and urinary symptoms) in PD patients. Although there are side effects from LCIG, close and careful observation can help in improving NMS.

LCIG provides an uninterrupted intestinal levodopa infusion by percutaneous endoscopic gastrojejunostomy (PEG‐J). Thus, by decreasing the fluctuations in plasma concentrations of levodopa, LCIG may reduce the motor fluctuations and NMS burden in advanced PD. Further, it is important to mention that dose modification and individualization of therapy are essential for optimal effect in PD patients.

## CONFLICTS OF INTEREST

Walaa A. Kamel and Jasem Y. Al‐Hashel have received honoraria for research support from AbbVie.

## AUTHORS' CONTRIBUTION

Both the authors contributed to conception, organization, and execution. Both the authors contributed to manuscript drafting and revision. All authors approved the final version of the manuscript and agree to be accountable for the content of the work.

## AFFIRMATION

The authors confirm that the work is consistent with the journal ethical guidelines.

### Peer Review

The peer review history for this article is available at https://publons.com/publon/10.1002/brb3.1757.
